# Isolation and characterisation of a human anti-idiotypic scFv used as a surrogate tumour antigen to elicit an anti-HER-2/neu humoral response in mice

**DOI:** 10.1038/sj.bjc.6601825

**Published:** 2004-04-27

**Authors:** M Coelho, P Gauthier, M Pugnière, F Roquet, A Pèlegrin, I Navarro-Teulon

**Affiliations:** 1 Tumour Immunotargeting and Antibody Engineering, INSERM, EMI0227, 34298 Montpellier, France; 2 Center for Pharmacology and Health Biotechnology, CNRS, UMR 5160, 34093 Montpellier, France

**Keywords:** anti-idiotypic antibody, HER-2/neu, vaccine, active immunotherapy

## Abstract

HER-2/neu is a tumour antigen that is overexpressed in human breast tumours. Among the vaccine strategies developed to overcome immune tolerance to self-proteins, vaccination with anti-idiotypic (anti-Id) antibodies has been described as a promising approach for treatment of several malignant diseases. To develop an active immunotherapy for cancer patients positive for HER-2/neu, we investigated immunisation with human anti-Id single-chain fragments (scFv) mimicking the conformation of HER-2/neu protein to induce a humoral response in mice. We selected by phage display two human anti-Id scFv (Ab2*β*) directed against trastuzumab F(ab′)_2_ fragments (Ab1), a humanised anti-HER-2/neu monoclonal antibody. Using competitive ELISA and Biacore biosensor analysis, we showed that anti-Id scFv 40 and scFv 69 could inhibit HER-2/neu binding to trastuzumab. Following vaccination of BALB/c mice with the soluble or phage-displayed scFv, Ab3 polyclonal antibodies, and among them Ab1′ antibodies able to bind HER-2/neu, were detected in the sera of the immunised mice. These results demonstrate that the human anti-Id scFv could act as a surrogate antigen for HER-2/neu. The present study strongly suggests that the novel 30 kDa human mini-antibody could be used as an anti-idiotype-based vaccine formulation to induce an effective humoral response in patients bearing HER-2/neu-positive tumours.

For active cancer immunotherapy, the tumour-associated antigen (TAA) HER-2/neu is a promising cancer vaccine candidate since it is overexpressed on the surface of a number of human cancers, but its expression in normal tissue is restricted. This 185-kDa transmembrane phosphoglycoprotein consists of a cysteine-rich extracellular domain (ECD) that functions in ligand binding and an intracellular cytoplasmic domain with kinase activity. Moreover, HER-2/neu is a member of the epidermal growth factor receptor family (Coussens *et al*, 1985; Bargmann *et al*, 1986). Cancers in which HER-2/neu protein overexpression occurs include human adenocarcinomas such as breast (in 20–40% of intraductal carcinomas) (Slamon *et al*, 1987) or ovary (in 30%) (Berchuck *et al*, 1990). Although overexpression has been linked to more aggressive disease and a poorer prognosis in node-positive breast cancer, it has also been established that it is related to a favourable prognosis in some patients with stage I breast tumours that contain inflammatory infiltrates which may represent an immune response directed against autologous cancer cells (Rilke *et al*, 1991). One hypothesis is that the better outcome in these patients may be related to the generation of an HER-2/neu-specific immune response which could directly or indirectly limit further growth and metastasis. Different investigations defining HER-2/neu-specific immunity in patients with cancer indicate that high levels of both T cell and antibody immunity exists in some patients, even if it is low or lacking in the majority of them (Disis *et al*, 1994; Fisk *et al*, 1995; Peoples *et al*, 1995). This strongly suggests that immunological tolerance exists, probably related to the oncofetal origin of HER-2/neu, and that it represents a barrier to effective vaccination against this antigen (Nanda and Sercarz, 1995; Disis *et al*, 1998). Furthermore, different studies have proven that anti-HER-2/neu monoclonal antibodies (mAbs) can be effective in eradicating tumours (Baselga and Albanell, 2001).

These findings have stimulated further studies to test vaccine strategies to induce and increase immunity to HER-2/neu for the treatment of breast cancer or for the prevention of recurrent disease. Effective vaccine strategies must circumvent tolerance. For this reason, methods to break this tolerance, such as presenting the critical epitope in a different molecular environment to the tolerised host, have been developed. Several studies have evaluated whether tolerance to HER-2/neu could be circumvented by immunisation with either peptide-based vaccines (Disis *et al*, 1996, 2002; Dakappagari *et al*, 2000) or DNA (Concetti *et al*, 1996; Di Carlo *et al*, 2001; Pilon *et al*, 2001). Some encouraging results were obtained but with persistence of problems such as the weak immunogenicity of the peptides or the potential adverse effect of administering plasmid DNA encoding a functional oncogene. In this context, the immune network hypothesis (Jerne, 1974) seems to offer a unique approach to transform epitope structures into Id determinants expressed on the surface of antibodies. Active immunisation with tumour-specific Id vaccines has very early been shown to inhibit tumour growth in animals (Kennedy *et al*, 1985). Carcinoembryonic antigen (CEA) mimicry by (i) an anti-Id single-chain variable fragment (scFv) constructed from a murine mAb (Tripathi *et al*, 1998) and (ii) murine dendritic cells pulsed with anti-Id antibody (Saha *et al*, 2003) were published with convincing results regarding the level of the humoral response raised in mice. Recently, a study from the same group also described the use of a murine monoclonal anti-Id antibody as a surrogate antigen for human HER-2/neu (Baral *et al*, 2001). In human trials, if the experience using anti-Id antibodies to stimulate immunity against tumours is limited, the results are promising with minimal observed toxicity. Anti-Id antibodies that functionally mimic an antigen have been used for the treatment of colorectal carcinoma (CEA, (Foon *et al*, 1999)), B-and T-cell lymphoma (gp72 (Kwak *et al*, 1992), and gp37 (Bhattacharya-Chatterjee *et al*, 1988), respectively), and for the treatment of melanoma by triggering an active immune response against the disialoganglioside GD2 (Foon *et al*, 2000). Anti-Id mAbs mimicking the human high molecular weight melanoma-associated antigen have been tested in a clinical trial to implement active specific immunotherapy (Mittelman *et al*, 1990).

Antibody-variable region fragments, obtained by recombinant DNA technology among which phage display methodology (Winter *et al*, 1994), have attracted considerable attention, since these products can be easily engineered for specific purposes such as therapy of cancer (Nilsson *et al* (2000) for a review).

Using the synthetic human ETH-2 library (Pini *et al*, 1998), we selected by phage display and characterised anti-Id scFv (Ab2) directed against the trastuzumab F(ab′)_2_ fragments (Ab1). Data obtained from an *in vivo* study using different immunisation schedules indicate that these human anti-Id scFv can induce an effective anti-HER-2/neu humoral response in experimental animals and suggest that they could serve as a surrogate for the tumour antigen HER-2/neu.

## MATERIALS AND METHODS

### Materials

The human ovarian SK-OV-3 cell line, which overexpresses HER-2/neu, and the hamster ovarian CHO cell line were obtained from American Type Culture Collection (Rockville, MD, USA). All the *in vivo* experiments were performed in compliance with the French guidelines for experimental animal studies (Agreement No. A34220) and fulfil the UKCCCR Guidelines for the welfare of animals in experimental research. Six-to eight-week-old female BALB/c mice were obtained from Iffa Credo (L'arbresle, France). The ETH-2 synthetic human antibody phage library in the scFv format (Pini *et al*, 1998) was kindly provided by Dr D Neri (ETH Zurich, Switzerland). Anti-HER-2/neu monoclonal antibody FRP5 was kindly provided by Professor N Hynes (FMI, Basel, Switzerland). Trastuzumab was purchased from Roche (F Hoffmann-La Roche Ltd, Bazel, Switzerland). The human IgG1, used as a control, was kindly provided by Mabgène (Alès, France). HER-2/neu ECD-Fc fusion protein, composed of two extracellular domains of the receptor linked by a human Fc fragment, was kindly provided by Professor JP Mach (Biochemistry Institute, University of Lausanne, Switzerland). Human IgG1 control and trastuzumab F(ab′)_2_ fragments were obtained by pepsin digestion (Sigma, St Louis, MO, USA), at 37°C for 4 h, at a 3% (w w^−1^) ratio of pepsin/IgG in appropriate buffer (0.2 M sodium acetate, pH 4). The antibody fragments were purified by gel-filtration chromatography on a Sephacryl 100 column (Amersham Biosciences, Buckinghamshire, UK). SDS–PAGE (8.5%) revealed more than 95% purity of the deposited proteins.

### Phage display selection of anti-trastuzumab F(ab′)_2_ fragments anti-Id scFv antibodies (Ab2)

Protocols for phage display panning, and analysis were as previously described (Pini *et al*, 1998). The phage-displayed library was panned with trastuzumab F(ab′)_2_ fragments in three sequential rounds of panning on immunotubes. Briefly, 5 × 10^12^ phages were added to tubes, coated with trastuzumab F(ab′)_2_ fragments at a concentration of, respectively, 100, 50 and 10 *μ*g ml^−1^ in PBS for all three rounds. Phage pools were assessed by ELISA. In all, 96-well microtitre plates were coated overnight at 4°C with 0.5 *μ*g protein per well with either trastuzumab or human IgG1 F(ab′)_2_ fragments or anti-fd phage Ig (Sigma) in PBS. Plates were then blocked for 2 h at 37°C with PBS, 2% non fat-dried milk. A volume of 100 *μ*l of serial dilutions of polyclonal phage solutions in PBS was added to each well. Plates were incubated at room temperature for 1.5 h. Bound phage-scFv were detected by reaction for 1 h 30 min at 37°C with a horseradish peroxidase (HRP)-conjugated anti-M13 mAb (Amersham Biosciences). The plates were developed using *o*-phenylenediamine substrate (OPD) according to the supplier's instructions (Sigma).

### Production and purification of anti-trastuzumab F(ab′)_2_ fragments soluble anti-Id scFv

After the third round of selection, *Escherichia coli* HB2151 cells were infected with the phages to produce soluble antibodies rather than phage-bound antibodies. Single ampicillin-resistant infected *E. coli* HB2151 colonies were picked in 96-well tissue culture plates and grown for 3 h at 37°C in 2 × TY, 100 *μ*g ml^−1^ ampicillin, 0.1% glucose. ScFv expression was induced with isopropyl *β*-D-thiogalactopyranoside (IPTG) to a final concentration of 1 mM for 16 h at 30°C. The bacterial supernatants were evaluated for reactivity with trastuzumab or human IgG1 F(ab′)_2_ fragments by ELISA. The ELISA was performed as described above except that after incubation of *E. coli* HB2151 supernatants, bound soluble scFv was detected using HRP-conjugated anti-FLAG-tag M2 Ab (Sigma) for 1.5 h.

For Western blot analysis, protein extracts were size fractionated by SDS–PAGE (12.5%) and electroblotted onto nitrocellulose. The blot was blocked at room temperature with shaking for 1 h in TBS, 1% nonfat-dried milk, and probed at room temperature for 2 h with the detecting Ab (HRP-conjugated anti-FLAG-tag M2) in TBS, 5% skimmed dried milk. The blot was washed three times and developed using 4-chloro-1-naphthol as substrate (Sigma).

For each positive clone, 15 *μ*g of plasmid DNA was purified using the NucleoSpin® Plasmid kit (Macherey-Nagel, Düren, Germany). Sequencing reactions were carried out using the Dye Terminator Sequencing kit for automatic determination of sequences with ABI PRISM 377 (Applied Biosystems, Foster City, CA, USA). The primers 5′-TACTACGCAGACTCCGTGAAG-3′ and 5′-GAATTTTCTGTATGAGG-3′ were used for annealing.

For high-scale bacterial expression and purification of scFv 39, 40 and 69, overnight cultures of *E. coli* HB2151 single colonies were grown in 1 l 2 × TY, 100 *μ*g ml^−1^ ampicillin, 0.1% glucose, at 37°C. The expression of the scFv was induced for 16 h at 30°C by adding 1 mM IPTG at 0.6 OD_600_ cultures. Bacterial pellets were resuspended in lysis buffer (30 mM Tris-HCl, 20% sucrose, 1 mM EDTA, 1% PMSF, pH 7) and cooled on ice for 30 min. After centrifugation, the supernatant (periplasmic fraction) was filtered through a 0.45-*μ*m filter. Clear supernatant from each culture was purified using IMAC (Casey *et al*, 1995). ScFv were loaded onto a 5-ml Hitrap Ni-activated Chelating HP column (Amersham Biosciences) in loading buffer (0.02 M sodium phosphate, 0.5 M NaCl, pH 7.4) and were eluted by applying a 25 ml 0–0.5 M imidazole gradient. ScFv-containing fractions were pooled and concentrated to a volume <1 ml on Amicon Ultra 30 000 MWCO (Millipore, Bedford, MA, USA). Additional purification was achieved by fast protein liquid chromatography on a Superdex 75 gel-filtration column (Amersham Biosciences). The purity of the recombinant proteins, rescued in PBS, was evaluated on SDS–PAGE (12.5%). Protein bands were detected by silver staining.

### *In vitro* characterisation of anti-Id scFv by ELISA and BIACORE

To characterise the anti-Id scFv, competitive ELISA experiments were carried out using either HER-2/neu ECD-Fc fusion protein to inhibit the binding of soluble scFv to trastuzumab F(ab′)_2_ fragments (Ab1) or purified scFv to block the binding of HER-2/neu ECD-Fc fusion protein to Ab1. Each soluble scFv was used at a dilution giving an *A*_490_ of 1–1.5 in ELISA. An irrelevant soluble scFv, named T1, isolated from the ETH-2 library and which does not bind to trastuzumab F(ab′)_2_ fragments, was used as a control (data not shown). In the first format, serial dilutions of HER-2/neu ECD-Fc fusion protein, ranging in concentration from 25 to 0 *μ*g ml^−1^, were used as inhibitor solutions; bound soluble scFv was detected as previously described. Recombinant human carcinoembryonic antigen (rhCEA) was used as an irrelevant inhibitor. This format of inhibition was also used immediately after the third round of selection to check the inhibition of the binding of individual periplasmic fractions on trastuzumab F(ab′)_2_ fragments by HER-2/neu ECD-Fc fusion protein. In the second format, competition was performed using various concentrations of purified soluble scFv ranging from 250 to 0 *μ*g ml^−1^ solutions and HER-2/neu ECD-Fc fusion protein at a dilution giving an *A*_490_ of 1–1.5 in ELISA. Bound HER-2/neu ECD-Fc fusion protein was subsequently detected with HRP-conjugated anti-HER-2/neu FRP5 mAb.

Binding experiments of anti-trastuzumab F(ab′)_2_ fragments scFv and HER-2/neu ECD-Fc fusion protein on trastuzumab F(ab′)_2_ fragments were performed at 25°C by SPR analysis using a BIACORE 2000 instrument (Biacore AB, Uppsala, Sweden). Trastuzumab F(ab′)_2_ fragments were covalently immobilised on a CM5 sensor chip surface using the amine coupling method according to the manufacturer's instructions (Biacore AB). A control reference surface was prepared using the same chemical treatment of the flow cell surface without injection of trastuzumab F(ab′)_2_ fragments. HER-2/neu ECD-Fc fusion protein (15 *μ*g ml^−1^) and soluble scFv (50 *μ*g ml^−1^) in HBS-EP buffer (10 mM HEPES, 3 mM EDTA, 150 mM NaCl, 0.005% P20, pH 7.4) were injected over the flow cell and the dissociation phase was followed by a regeneration step (10 *μ*l 100 mM HCl). The flow rate was 50 *μ*l min^−1^. All the sensorgrams were corrected by subtracting the low signal of the control reference surface. The BIA evaluation 3.2 software was used to fit the data using a 1 : 1 Langmuir global model. For the inhibition experiments, a first injection of anti-Id scFv 69 (50 *μ*l at 2 *μ*M) was followed by a second injection of HER-2/neu ECD-Fc fusion protein (50 *μ*l at 0.06 *μ*M). After a regeneration step with 10 *μ*l of 100 mM HCl, a similar experiment was performed with a first injection of buffer instead of the scFv. The flow rate was 10 *μ*l min^−1^. To evaluate the percent inhibition, we injected the anti-Id scFv 69 followed by a long dissociation step. This dissociation curve was then substracted from the HER-2/neu association curve.

### Analysis of the Ab3 response in mouse sera

Two immunisation schedules were carried out. In the first one (protocol 1), five 6- to 8-week-old female BALB/c mice per group were immunised i.p. monthly for 3 months with 50 *μ*g of either purified soluble anti-Id scFv 40 or 69, 20 *μ*g of HER-2/neu ECD-Fc fusion protein in 100 *μ*l PBS (negative control group). Injections were performed after emulsion with CFA for the first immunisation and with IFA for subsequent ones. In the second protocol, inspired by the work of Magliani *et al* (1998), 6-to 8-week-old female BALB/c mice were immunised four times. A first s.c. injection of either 50 *μ*l of freshly prepared phage-scFv 40 or 69 at a titre of 10^14^ TU ml^−1^, 20 *μ*g of HER-2/neu ECD-Fc fusion protein, or PBS was performed after emulsion with CFA. This injection was followed 2 weeks later by a second s.c. administration of the same dose with IFA. Two additional injections were given i.p. at 21 and 35 days after the initial immunisation (protocol 2). For serum antibody measurement, mice were bled and sera were drawn from the tail vein and stored at −20°C for assay. The sera were checked for anti-anti-Id (Ab3) responses by flow cytometry and ELISA on the HER-2/neu ECD-Fc fusion antigen as described below. Analysis of the results obtained was also performed according to the method described by Magliani *et al* (1998). A positive control consisting of pooled sera from mice immunised with HER-2/neu ECD-Fc fusion protein was included in each determination. Results were expressed in arbitrary units as the ratio between absorbance of the test serum to 70% of the absorbance of the positive control serum multiplied by 1000. The 30%, subtracted from the positive control serum absorbance, was due to the anti-Fc response (see Results).

To detect anti-anti-Id scFv in mouse sera, an ELISA was performed by coating 96-well plates with soluble anti-Id scFv at 5 *μ*g ml^−1^ and then washing and blocking with PBS, 1% BSA for 2 h at 37°C. Serial dilutions of sera from immunised mice (1 to 1 : 3200 in PBS, 0.5% BSA) were incubated for 1.5 h at room temperature. Bound IgG was detected after incubation for 1.5 h of a HRP-conjugated goat anti-mouse IgG (Sigma).

To further confirm the presence of anti-anti-Id scFv, an inhibition ELISA was performed by preincubating serial dilutions of sera from immunised mice (1 to 1 : 640 in PBS, 0.5% BSA) with soluble scFv for 2 h at 37°C. Next, 100 *μ*l of each mixture was added to the wells, coated with soluble anti-Id scFv. The plates were incubated at room temperature for 1.5 h. Bound scFv were detected as previously described.

### ELISA and FACS analysis of anti-HER-2/neu antibodies (Ab1′) in mouse sera

Indirect ELISA was performed by coating 96-well plates overnight at 4°C with HER-2/neu ECD-Fc fusion protein at 2 *μ*g ml^−1^ and then blocked with PBS, 1% BSA. A volume of 100 *μ*l of diluted sera from mice (1 : 100 in PBS, 0.5% BSA) were added in each well and incubated for 1.5 h at room temperature. Then, 100 *μ*l of either goat anti-mouse IgG (whole molecule)-HRP conjugate, goat anti-mouse IgG (*γ*-chain specific)-HRP conjugate, or goat anti-mouse IgM (*μ*-chain specific)-HRP conjugate was added, incubated for 1.5 h, and detected as described above.

For FACS analysis, HER-2/neu-positive SK-OV-3 and HER-2/neu-negative CHO cell lines were incubated for 1 h at 4°C with either 100 *μ*l of each mouse serum or 100 *μ*l trastuzumab diluted in PBS, 1% BSA, 1 mg ml^−1^ azide. After washing, the cells were incubated for another 45 min at 4°C with either 100 *μ*l of diluted sheep anti-mouse IgG-FITC-labelled antibody for sera or anti-human-FITC-labelled antibody (Sigma) for trastuzumab. The stained cells were suspended in 500 *μ*l PBS, and stored at 4°C before FACScan analysis (Becton Dickinson, Franklin Lakes, MD, USA).

## RESULTS

### Isolation of an anti-Id scFv

**Figure 1:**
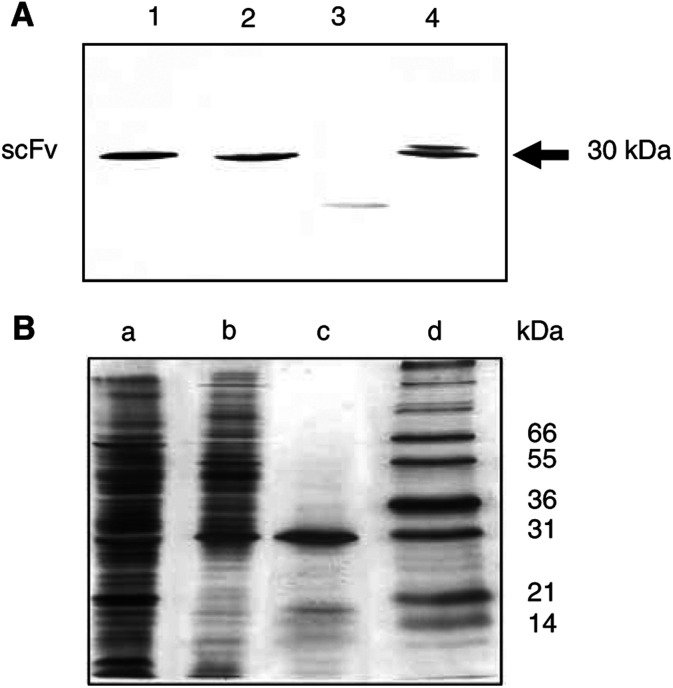
Analysis of antibody-containing periplasmic fractions containing antibodies (**A**) After induction of single ampicillin-resistant infected *E. coli* HB2151 colonies with 1 mM IPTG. Lane 1: clone 39; lane 2: clone 40; lane 3: clone 92; lane 4: clone 69. After size fractionation on 12.5% SDS–PAGE, protein extracts were blotted onto nitrocellulose. The immunoblot was developed with HRP-conjugated M2 anti-FLAG mAb (1 : 2000) followed by addition of the 4-chloro-1-naphtol substrate. (**B**) Before and after purification. The purity was controlled on 12.5% SDS–PAGE gel followed by silver staining. Lane a: nonpurified periplasmic fraction. Lane b: Periplasmic fraction purified on a Hitrap Ni-activated chelating column. Lane c: Periplasmic fraction purified on a Hitrap Ni-activated chelating column followed by gel filtration on Superdex 75. Lane d: Standard molecular mass markers.

The selection of the anti-Id scFv, performed by three rounds of panning of the synthetic ETH-2 scFv library on immobilised trastuzumab F(ab′)_2_ fragments (Ab1), resulted in a substantial affinity enrichment of the library on either trastuzumab or human IgG1 F(ab′)_2_ fragments (used as a control) as checked by ELISA using the recombinant polyclonal phage supernatants. A 2 × 10^4^-fold increase of phage titre between the first and the third round of selection was also observed. After cloning, antibody supernatants derived from 96 induced individual clones of the third round were then used to infect *E. coli* HB2151 to produce soluble scFv antibodies. In total, 96 periplasmic fractions were tested for their reactivity with human IgG1 or trastuzumab F(ab′)_2_ fragments by ELISA. The ratio between the number of phages specific for trastuzumab and those reacting with irrelevant (Fab′)_2_ IgG was about 30% after the third round of selection. Western blot analysis of the positive periplasmic fractions led us to select scFv antibodies consisting of single monomers with the approximate molecular mass of about 30 kDa and to exclude those with a lower molecular mass (Figure 1A

). The selected periplasmic fractions were checked by ELISA for inhibition of their binding on trastuzumab F(ab′)_2_ fragments by either HER-2/neu ECD-Fc fusion protein or rhCEA (recombinant human CEA used as an irrelevant inhibitor). Only clones showing marked inhibition (>50%) were further analysed. After DNA sequence determination of the V_H_ and V_L_ regions of the antibodies, three unique scFv were selected (Table 1

**Table 1:** Predicted amino-acid sequences of the CDR3 regions of V_H_ and V_L_ domains of the three unique scFv 39, 40 and 69

**scFv**	**H-CDR3**	**L-CDR3**
39	CAK**KKIGP**FDY	NSS**NSSPRPNAP**VVF
40	CAK**NYQIHP**FDY	NSS**DPDQLL**VVF
69	CAK**NVHIQP**FDY	NSS**EPTPPR**VVF

a Amino-acid sequences are given in the one letter code. Random loops of 5 or 6 amino acids introduced at position 95 of V_H_ or V_L_ are in bold characters.

). The three antibodies exhibited different random loops introduced at position 95 of V_H_ or V_L_ and used germline gene segments corresponding to DP-47 for the heavy chain and DPL-16 for the light chain.

Anti-Id scFv 39, 40 and 69 were purified to 90% homogeneity using immobilised metal ion affinity chromatography (IMAC) followed by gel filtration on a Superdex 75 as shown in Figure 1B for anti-Id scFv 69. Anti-Id scFv 39 and 40 were obtained with the same level of purity. These three scFv were also expressed, displayed on phage after infection of *E. coli* TG1, and the immunoreactivity of each phage solution controlled by ELISA on trastuzumab F(ab′)_2_ fragments (data not shown).

### *In vitro* characterisation of the Ab2 anti-Id scFv

**Figure 2:**
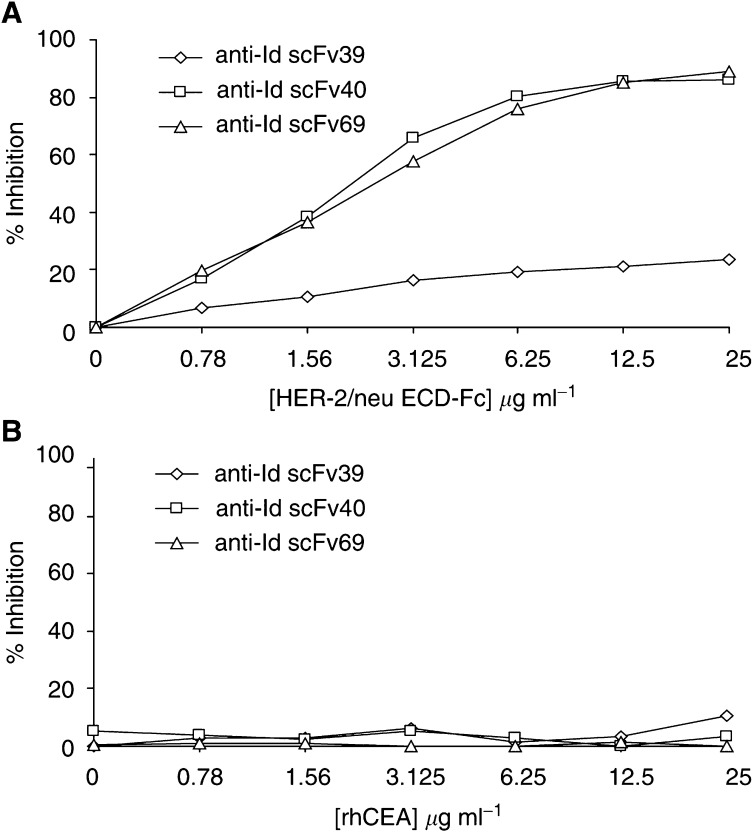
Inhibition of purified soluble scFv 39, 40 and 69 binding on trastuzumab F(ab′)_2_ fragments by (**A**) HER-2/neu ECD-Fc fusion protein and (**B**) rhCEA, using a competitive ELISA. Increasing amounts of inhibitor were mixed with either anti-Id scFv 39, 40 or 69 used at a dilution giving an *A*_490_ ranging from 1 to 1.5 in ELISA (corresponding to a concentration of about 20 *μ*g ml^−1^). The incubation on trastuzumab F(ab′)2 fragments for 1.5 h was followed by the detection of scFv binding with HRP-conjugated M2 anti-FLAG mAb (1 : 2000). The results obtained are expressed as percent inhibition at each concentration of inhibitor.

**Figure 3:**
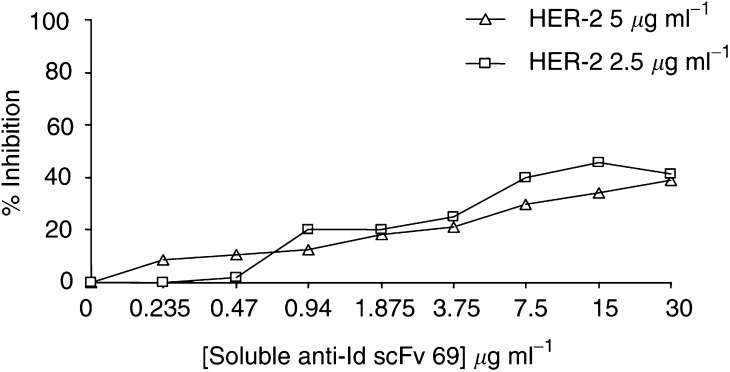
Inhibition of HER-2/neu ECD-Fc fusion protein binding on trastuzumab F(ab′)_2_ fragments by purified scFv 69 by competitive ELISA. Increasing amounts of inhibitor were mixed with HER-2/neu ECD-Fc fusion protein at 2.5 or 5 *μ*g ml^−1^ and incubated with trastuzumab F(ab′)_2_ fragments for 1.5 h. HER-2/neu ECD-Fc fusion protein binding was then detected with peroxidase-conjugated anti-HER-2/neu FRP5 mAb (1 : 3000). The results obtained are expressed as percent inhibition at each concentration of inhibitor.

To characterise the binding properties of the anti-Id scFv, we first checked whether or not these scFv exhibited competitive binding with trastuzumab F(ab′)_2_ fragments (Ab1) for HER-2/neu epitopes. When the HER-2/neu ECD-Fc fusion protein was used as an inhibitor, the binding of both anti-Id scFv 40 and 69 on trastuzumab F(ab′)_2_ fragments was inhibited by approximately 90% (Figure 2A

). The binding of anti-Id scFv 39 was only inhibited by 23%; thus, anti-Id scFv 39 was not used for subsequent experiments. The same competition experiment was performed using rhCEA (185 kDa) (Figure 2B). Inhibition curves showed that even at high concentrations of rhCEA, this antigen (whose molecular mass is nearly the same as the HER-2/neu ECD-Fc fusion protein) was unable to compete with anti-Id scFv for the binding on trastuzumab F(ab′)_2_ fragments. Anti-Id scFv 40 and 69 were subsequently used to inhibit the binding of HER-2/neu ECD-Fc fusion protein at two different concentrations on trastuzumab F(ab′)_2_ fragments. In this format, the maximum inhibition observed was nearly 40% for anti-Id scFv 69 at 30 *μ*g ml^−1^ (Figure 3

) and even with concentrations up to 250 *μ*g ml^−1^ (data not shown). The same result was obtained for anti-Id scFv 40 (data not shown).

**Figure 4:**
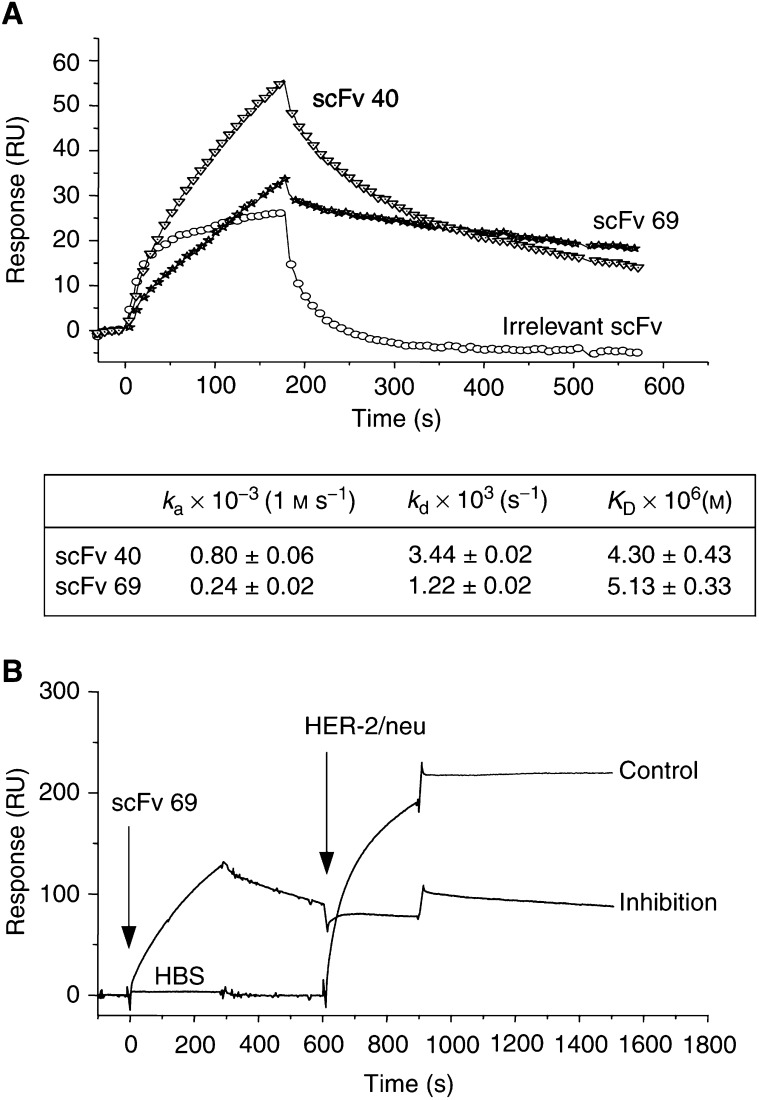
BIACORE experiments: (**A**) binding kinetics of anti-Id scFv 40, 69 and irrelevant scFv on trastuzumab F(abi′)_2_ fragments immobilised on a CM5 sensor chip and equilibrium affinity constants (inserted Table), (**B**) inhibition of HER-2/neu ECD-Fc fusion protein binding on trastuzumab F(ab′)_2_ fragments immobilised on a CM5 sensorchip by anti-Id scFv 69.

Binding kinetic parameters of anti-Id scFv 40 and 69 on immobilised trastuzumab F(ab′)_2_ fragments were measured using surface plasmon resonance (SPR) technology (Figure 4A

). Whereas the affinity constants of both scFv were in the same order of magnitude (5 *μ*M), anti-Id scFv 40 showed higher association and dissociation rates than those of scFv 69 (Figure 4A). Under the same conditions, the affinity constant of HER-2/neu ECD-Fc fusion protein was in the nanomolar range (*K*_D_=2.9±0.1 nM). The inhibition of the binding of HER-2/neu ECD-Fc fusion protein on immobilised trastuzumab F(ab′)_2_ fragments by anti-Id scFv 69 was also evaluated using this technology. The prior injection of anti-Id scFv 69 inhibited by 83% the binding of HER-2/neu ECD-Fc fusion protein on trastuzumab F(ab′)_2_ fragments (Figure 4B). These results confirmed the data obtained by competitive ELISA and gave an even higher percent inhibition, suggesting that anti-Id scFv 40 and 69 could be true Ab2*β*. If so, these scFv should be able to induce a polyclonal anti-anti-Id response (Ab1′) when injected into mice.

### Immunising properties of anti-Id scFv 40 and anti-Id scFv 69

**Figure 5:**
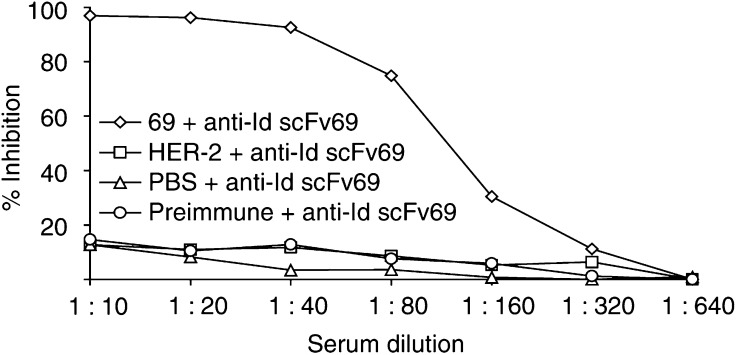
Analysis of the Ab3 anti-anti-Id scFv 69 response in sera of BALB/c mice (immunisation protocol 1) by inhibition of the binding of anti-Id scFv 69 (Ab2) on immobilised trastuzumab F(ab′)_2_ fragments (Ab1) by inhibition ELISA. Serial dilutions of preimmune sera or sera from mice each of the three groups: primed with HER-2/neu ECD-Fc fusion protein or with PBS or with anti-Id scFv 69 were preincubated with soluble anti-Id scFv 69. This solution was subsequently incubated for 2 h on trastuzumab F(ab′)_2_ fragments followed by the detection of bound scFv by HRP-conjugated M2 anti-FLAG mAb. The results obtained are expressed as percent inhibition at each serum dilution.

Six-to eight-week-old female BALB/c mice were immunised either i.p. with soluble purified anti-Id scFv 40 and 69 (protocol 1) or s.c. with scFv displayed on phages (protocol 2). Sera of the five mice in each group were individually tested by ELISA for their reactivity against Ab2 immunogens (anti-Id scFv 40 or 69) after three or four injections. All the mice developed significant immunity against anti-Id, and the titres were further increased with further immunisations in both protocols (see Sect. 2.4). Preimmune sera or control sera of mice immunised with PBS were tested negative against anti-Id scFv 40 and anti-Id scFv 69. No specific response was observed either in sera of mice injected with wild-type phages (data not shown). To demonstrate the specificity of the Ab3 response against anti-Id scFv, an inhibition ELISA was carried out by inhibiting the binding of each anti-Id scFv on trastuzumab F(ab′)_2_ fragments by serial dilutions of sera from immunised mice. A 1 : 20 dilution of immune serum from a mouse immunised with anti-Id scFv 69 displayed on phage particles inhibited by nearly 100% Ab2 (anti-Id scFv 69) binding to Ab1 (trastuzumab F(ab′)_2_ fragments), suggesting that antibodies contained in the sera were true anti-anti-Id in nature (Figure 5

). Anti-Id scFv 40 gave similar results (data not shown). In contrast, no inhibition was observed with immune sera from mice immunised with HER-2/neu ECD-Fc fusion protein or PBS.

### Idiotype analysis of Ab3 (Ab1′) induced by immunisation with anti-Id scFv

**Figure 6:**
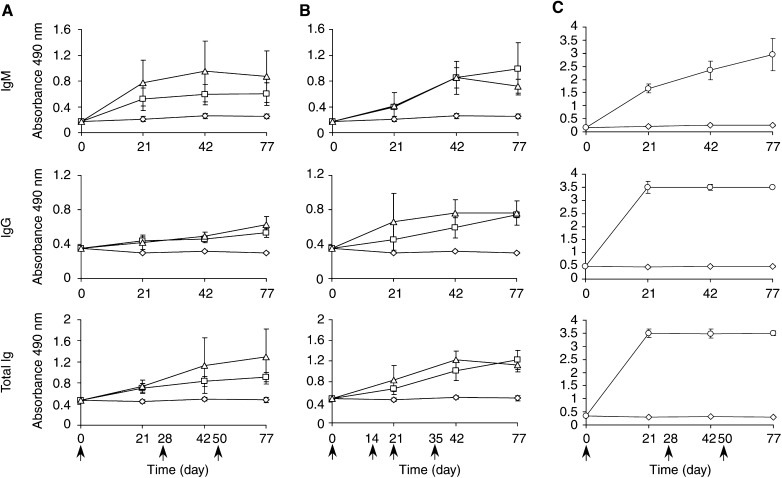
Analysis of the Ab1′ response in sera of mice. Determination of anti-HER-2/neu IgG or IgM or total Ig levels in sera collected at various times after immunisation with (**A**) soluble purified anti-Id scFv, protocol 1; (**B**) phage-displayed scFv, protocol 2; (**C**) HER-2/neu ECD-Fc fusion protein. Mice were immunised with these antigens at the indicated times (arrows). Bound antibodies in sera diluted 1 : 100 were detected with HRP-conjugated anti-total Ig or *μ*-or *γ*-chain-specific anti-mouse Ig. Data are presented as mean±s.d. *A*_490_ values of five determinations, corresponding to the five mice in each group. Curves indicate sera from mice immunised with PBS (⋄), sera from mice immunised with anti-Id scFv 40 (□), sera from mice immunised with anti-Id scFv 69 (▵), and sera from mice immunised with HER-2/neu ECD-Fc (○).

To detect the subset of antibodies called Ab1′, able to bind to the target antigen, ELISA were performed on HER-2/neu ECD-Fc fusion protein using mouse sera diluted 1 : 100. The use of different HRP-conjugated Ig allowed us to determine the IgG, IgM, and total Ig levels induced in mouse sera by immunisation with anti-Id scFv and control immunogens (Figure 6

). The absorbance values plotted at each time correspond to the mean value for each group of five mice±standard deviations (s.d.). These results demonstrated that (i) patterns of induction of IgG or IgM anti-HER-2/neu antibodies were similar for the two immunisation protocols (Figure 6A, B); (ii) a significant portion of the polyclonal Ab3 reacted with the antigen (Figure 6A, B), the level of Ab1′ reaching a plateau after three or four injections of immunogen; and (iii) as shown in Figure 6C, the titres of IgM as well as IgG anti-HER-2/neu ECD-Fc fusion protein response were, as expected, very high in the sera of mice immunised with HER-2/neu ECD-Fc fusion protein, whereas there was no reactivity in the negative control. In the sera of mice immunised with HER-2/neu ECD-Fc fusion protein, part of the very high titre against the adsorbed protein was due to an anti-human Fc response as shown when the reactivity of these sera against another recombinant Fc fusion protein, the so-called MICA-Fc, (kindly provided by Dr W Held, LICR, Lausanne Branch) was checked by ELISA. The data obtained (not shown) led us to conclude that the anti-Fc response was about 30% of the total response observed by ELISA on HER-2/neu ECD-Fc fusion protein.

### Flow cytometric analysis of Ab1′

**Figure 7:**
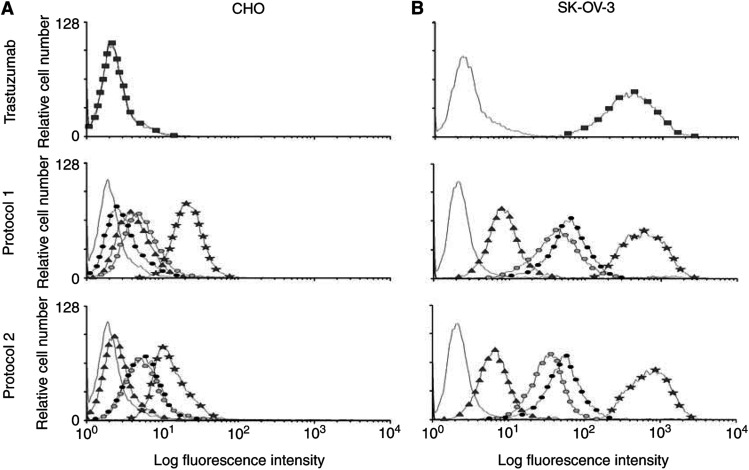
Analysis of the binding of mouse sera to cells after the two immunisation schedules: protocol 1 with soluble anti-Id scFv 40 and 69 and protocol 2 with phage-displayed scFv. The binding experiments were performed either on (**A**) CHO cells which do not express the HER2/neu receptor or (**B**) SK-OV-3-cells overexpressing HER2/neu receptor. The binding of antibodies was detected by FACS analysis using an FITC-labelled goat anti-mouse antibody. All antibody binding experiments were performed in comparison with trastuzumab detected using a FITC-labelled anti-human antibody and with the background fluorescence determined by binding on cells of the FITC-labelled secondary antibody alone. Curves indicate background fluorescence (solid line), control mAb trastuzumab (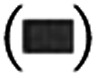), sera from mice immunised with PBS (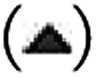), sera from mice immunised with anti-Id scFv 40 (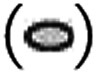), sera from mice immunised with anti-Id scFv 69 (•), and sera from mice immunised with HER-2/neu ECD-Fc (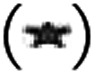 ).

To further confirm the nature of the Ab1′ induced by injections with anti-Id scFv 40 and 69, flow cytometric analyses of HER-2/neu-positive SK-OV-3 cells and HER-2/neu-negative CHO cells were performed with sera (1 : 100 dilution) from immunised BALB/c mice. Using trastuzumab mAb, both HER-2/neu overexpression at the surface of SK-OV-3 cells and the lack of expression of the receptor on the CHO cells surface were first confirmed (Figure 7A, B

). Then, we demonstrated that antibodies not only in sera from mice immunised with HER-2/neu ECD-Fc fusion protein but also in sera from mice immunised either with anti-Id scFv 40 or anti-Id scFv 69, specifically stained the SK-OV-3 cells. Only a weak reactivity was observed on CHO cell line. Representative results obtained at the end of the two immunisation schedules are presented also in Figure 7A, B. The fluorescence intensity increase on SK-OV-3 cells, as compared with CHO cells, were about 100, 25 and 2 for sera from mice immunised with HER-2/neu ECD-Fc fusion protein, anti-Id scFv 40 or 69 and PBS, respectively. The staining of cells obtained by using sera from mice injected with PBS was equivalent to that of preimmune sera, thus corresponding to the background signal. On the basis of these experiments, we concluded that true Ab1′ antibodies, which are specifically directed against the HER-2/neu receptor, were present in the sera of mice immunised with anti-Id scFv 40 and 69.

## DISCUSSION

The HER-2/neu oncogenic protein is a well-characterised TAA (Shepard *et al*, 1991). The main problem when using the HER-2/neu oncoprotein as a target antigen for active immunotherapy is immune tolerance to this self-antigen. Ab1 therapy as well as peptide or protein-based vaccines have been reported (Pegram *et al*, 1998; Disis *et al*, 2002). All the studies have pointed out the necessity to pursue the search for effective therapeutic approaches.

The present study was conducted to further explore the efficacy of the anti-Id (Ab2) strategy to generate an anti-HER-2/neu immune response. Trastuzumab, a humanised mAb specific for HER-2/neu, whose efficiency has been proven both alone and in combination with various chemotherapeutic agents in women with HER-2-positive metastatic breast cancer (Cobleigh *et al*, 1999; Slamon *et al*, 2001), was chosen as Ab1. Several authors have pointed out that a distortion of the scFv derived from a monoclonal anti-Id antibody or the differential orientation of the V_H_ or V_L_ domain in a scFv construct are likely to affect its antigen mimicry (Francisco *et al*, 1995; Tripathi *et al*, 1998). For this reason, the phage display technology was chosen to isolate human anti-Id scFv fragments from the synthetic ETH-2 library (Pini *et al*, 1998) since (i) such scFv do not need further engineering and (ii) they could be used for repeated immunisations of cancer patients without invoking a human anti-mouse antibody response.

The affinity selection was performed using proteolytically cleaved F(ab′)_2_ fragments of trastuzumab to avoid the selection of scFv specific for the Fc portion of the human IgG1. By using a conventional selection and elution method, we obtained specific clones with a diversity of 15%. Such a diversity, which is expected using this technique, could probably be improved by the method recently described by Goletz *et al* (2002), which involves specific elution with the antigen followed by trypsin treatment of eluted phages for generating large diversities of anti-Id single-chain antibody fragments from nonimmunised phagemid libraries using phage display. In our study, after the three rounds of panning, the selection procedure was based first on the direct binding of periplasmic fractions from individual *E. coli* HB2151 colonies to trastuzumab, their failure to bind to control IgG1, and secondly on a competitive ELISA using the antigen as inhibitor. Only clones whose binding to trastuzumab was markedly inhibited by soluble HER-2/neu ECD-Fc fusion protein (and not by rhCEA) were conserved for higher scale production and purification. Competitive ELISA was subsequently performed by inhibiting antigen binding to Ab1 using the selected scFv fragments. This strategy allowed us to isolate three fragments, termed scFv 39, 40 and 69, which recognised the binding site of Ab1 but exhibited different random loops of five or six amino acids in the CDR3 of the V_H_ and V_L_ domains. The analysis of these sequences showed that random loops appended in H-CDR3 (the largest and most diverse loop of the antigen recognition site of the antibody) for anti-Id scFv 40 and 69 presented 5/6 identical amino acids, whereas the H-CDR3 of anti-Id scFv 39 was quite different in sequence and appeared to be more positively charged. Concerning the L-CDR3, the three scFv used the DPL-16 germline gene segment, L-CDR3 of anti-Id scFv 39 differed from the others, especially from scFv 40, by a global positive charge. The L-CDR3 of scFv 69 seemed to be very highly constrained by the three proline residues in the loop.

As previously stated, two inhibition formats were used to demonstrate that the selected scFv could be true Ab2*β*. In the first one, HER-2/neu ECD-Fc completely inhibited the binding of purified soluble scFv 40 and 69 on trastuzumab. Owing to the partial inhibition observed for scFv 39, this fragment was not considered for subsequent experiments. When the ELISA was performed by inhibiting the binding of HER-2/neu antigen on trastuzumab using Ab2 scFv 40 or Ab2 scFv 69 as inhibitors, an inhibition of about 40% was observed. In this test, soluble scFv was used at concentrations up to 250 *μ*g ml^−1^ (giving an *A*490 nm ranging from 1 to 1.5 by ELISA), that is, 10 *μ*M, corresponding to a 1000-fold molar excess as compared with the soluble HER-2/neu ECD-Fc fusion antigen. This suggests that the lower inhibition observed in the second format (40 *vs* 90% in the first one) could not be attributed to the low inhibitor concentration. Most likely, the difference between the affinity constants for trastuzumab of anti-Id scFv 40 and 69 (in the *μ*M range) and that of HER-2/neu ECD-Fc fusion protein (in the nM range) could explain this result. By SPR technology, an 83% inhibition of the binding of the antigen on Ab1 by anti-Id scFv 69 was observed. This technology allowed us to vizualise the real-time kinetics of the reaction. Under these conditions, the dissociation of soluble scFv from immobilised trastuzumab was minimal, in contrast with the ELISA protocol where the incubation and washing steps could lead to dissociation due to the relatively low affinity of the scFv for trastuzumab. Furthermore, the percent inhibition determined by BIACORE was calculated by subtracting the dissociation level of soluble scFv from the association level of HER-2/neu ECD-Fc fusion protein.

At this stage of the study, anti-Id scFv 40 and 69 were predicted to be internal images of the tumour antigen HER-2/neu and supposed to induce polyclonal anti-anti-Id antibodies (Ab3) when injected into animals. Our vaccine strategy used anti-Id scFv 40 and 69 as immunogens either in a purified soluble form (protocol 1) or displayed on phage particles (protocol 2) and administrated by different routes. Based on the work of Galfre *et al* (1996), who obtained a strong antibody response in mice when phages were injected in physiological saline but found that the use of adjuvants resulted in higher titres especially for poorly immunogenic epitopes, we used Freund's adjuvants in both our protocols. At the end of the immunisation schedules, all the mice had developed an Ab3 response to the injected anti-Id scFv, as shown by the results of inhibition ELISA for the mice immunised with anti-Id scFv 69 (Figure 5). Among the Ab3 antibodies, the Ab1′ response is the only one clinically relevant since it is the only one to bind the target (Bhattacharya-Chatterjee *et al*, 1991). We indeed demonstrated an Ab1′ response by testing the immunoreactivity of each serum on HER-2/neu ECD-Fc fusion protein. A similar anti-HER-2/neu ECD-Fc fusion protein response was obtained in all immunised animals (with slight differences in titres within each group) after three or four immunisations with either 50 *μ*g purified anti-Id scFv 40 or anti-Id scFv 69 (protocol 1) or about 1 *μ*g anti-Id scFv 40 or anti-Id scFv 69 exhibited on phages (protocol 2). These results are in agreement with the findings of Galfre *et al* (1996), who showed an increased immunogenicity by using phages as ‘carriers’ throughout the immunisation schedule. No significant Ab1′ reactivity was detected in the sera of the negative control group injected with PBS. The Ab1′ nature of the induced antibodies was also studied by FACS analysis of the mouse sera. This technology revealed that Ab1 (trastuzumab) and Ab3 sera bound significantly to HER-2/neu-positive SK-OV-3 cells and confirmed the generation of true anti-anti-Id as well as HER-2/neu-specific antibodies using either soluble scFv or scFv displayed on phage particles (Figure 7). As expected, in the positive control group of mice injected with HER-2/neu ECD-Fc fusion protein, the observed immune anti-HER-2/neu response was higher than in the group injected with anti-Id scFv 40 and 69. It is important to remark that (i) this response would probably be much lower in humans since the extracellular domain of HER-2/neu is shed, circulates in the sera of patients and is available to the immune system for tolerance induction (Leitzel *et al*, 1992), (ii) the human Fc of the fusion protein is immunogenic in mice but not in humans and (iii) the immunogenicity of anti-Id scFv fragments could be increased by incorporating additional immunogenic sequences such as keyhole limpet haemocyanin (KLH) (Tripathi *et al*, 1998). Our results with regard to immunisation using anti-Id scFv, either in soluble form or displayed on phage, are in agreement with those of Magliani *et al* (1998) who described the use of anti-Id scFv antibody (soluble or phage-fused) to mimick the antigenic properties of the type III capsular polysaccharide of group B *Streptococcus*. As they did, we analysed the levels and types of antibodies raised in mice throughout the two immunisation schedules (data not shown). For immunisation protocol 1, the calculated mean values (in arbitrary units, see Sect. 4.5) for the levels of IgM, IgG and total Ig were at maximum 650, 250 and 530, respectively, as compared with 650, 300 and 700 in the work of Magliani *et al* (1998). Our results are thus similar and comparable to theirs.

Our data indicate that the 30-kDa human anti-Id scFv 40 and 69 can induce an effective anti-HER-2/neu humoral response in experimental animals and can serve as a surrogate for the tumour antigen HER-2/neu. As future prospects for our work, an active immunisation with anti-Id scFv 40 and 69 to prevent the development of tumours in a transgenic mouse model of HER-2/neu mammary tumorigenesis (as a model of human breast cancer) will be performed. This study will also permit us to further analyse induced T-cell specific antitumour responses as well as antibody-dependent cellular cytotoxicity. In experimental tumour models, anti-Id mimicking TAA were shown years ago to induce both B- and T-cell specific antitumour immune responses (Dunn *et al*, 1987; Nelson *et al*, 1987). Recently, Baral *et al* (2001) reported the use of a murine monoclonal anti-Id antibody as a surrogate antigen for human HER-2/neu. They demonstrated that the anti-anti-Id (Ab3) response generated against Ab2 in sera of immunised rabbits could serve an important secondary effector function by enhancing antitumour ADCC.

The human anti-Id scFv 40 and 69 should be useful tools to delineate the role of individual immune HER-2/neu specific antitumour responses and ultimately to develop strategies of human anti-Id-based vaccines for enhancing such immunity in patients bearing HER-2/neu-positive tumours.
